# A Novel Ingestion Strategy for Sodium Bicarbonate Supplementation in a Delayed-Release Form: a Randomised Crossover Study in Trained Males

**DOI:** 10.1186/s40798-019-0177-0

**Published:** 2019-01-24

**Authors:** Nathan Philip Hilton, Nicholas Keith Leach, S. Andy Sparks, Lewis Anthony Gough, Melissa May Craig, Sanjoy Kumar Deb, Lars Robert McNaughton

**Affiliations:** 1 Sports Nutrition and Performance Group, Department of Sport and Physical Activity, Edge Hill University, St Helens Road, Ormskirk, L39 4QP UK; 2 School of Health Sciences, Birmingham City University, Birmingham, UK; 3 Therapies Department, Liverpool Heart and Chest Hospital NHS Foundation Trust, Liverpool, UK; 4 School of Life Sciences, University of Westminster, London, UK; 5 Department of Sport and Movement Studies, Faculty of Health Science, University of Johannesburg, Johannesburg, South Africa

**Keywords:** Acid-base balance, Extracellular buffer, Bioavailability, Exercise-induced fatigue

## Abstract

**Background:**

Sodium bicarbonate (NaHCO_3_) is a well-established nutritional ergogenic aid, though gastrointestinal (GI) distress is a common side-effect. Delayed-release NaHCO_3_ may alleviate GI symptoms and enhance bicarbonate bioavailability following oral ingestion, although this has yet to be confirmed.

**Methods:**

In a randomised crossover design, pharmacokinetic responses and acid-base status were compared following two forms of NaHCO_3_, as were GI symptoms. Twelve trained healthy males (mean ± SD age 25.8 ± 4.5 years, maximal oxygen uptake ($\dot{\mathrm{V}}{\mathrm{O}}_{2\max }$) 58.9 ± 10.9 mL kg min^−1^, height 1.8 ± 0.1 m, body mass 82.3 ± 11.1 kg, fat-free mass 72.3 ± 10.0 kg) underwent a control (CON) condition and two experimental conditions: 300 mg kg^−1^ body mass NaHCO_3_ ingested as an aqueous solution (SOL) and encased in delayed-release capsules (CAP). Blood bicarbonate concentration, pH and base excess (BE) were measured in all conditions over 180 min, as were subjective GI symptom scores.

**Results:**

Incidences of GI symptoms and overall severity were significantly lower (mean difference = 45.1%, *P* < 0.0005 and 47.5%, *P* < 0.0005 for incidences and severity, respectively) with the CAP than with the SOL. Symptoms displayed increases at 40 to 80 min post-ingestion with the SOL that were negated with CAP (*P* < 0.05). Time to reach peak bicarbonate concentration, pH and BE were significantly longer with CAP than with the SOL.

**Conclusions:**

In summary, CAP can mitigate GI symptoms induced with SOL and should be ingested earlier to induce similar acid-base changes. Furthermore, CAP may be more ergogenic in those who experience severe GI distress with SOL, although this warrants further investigation.

## Key Points

- Delayed-release NaHCO_3_ mitigated GI distress compared with the aqueous solution ingestion form; therefore, athletes who have experienced problematic side-effects in the past may now benefit from supplementation.
- Time to reach peak blood bicarbonate increased with delayed-release NaHCO_3_, and therefore, requires earlier ingestion (~ 48 min) in comparison with the aqueous solution ingestion form.
- Bicarbonate bioavailability was enhanced in some individuals with delayed-release NaHCO_3_; hence, ingestion should be based upon individual concentration-time profiles in conjunction with GI symptoms.

## Background

Sodium bicarbonate (NaHCO_3_) is a well-established nutritional ergogenic aid. Supplementation can improve short-duration (~ 1–10 min), high-intensity exercise performance [1], with various meta-analyses confirming its efficacy [2–4]. As an extracellular buffering agent, NaHCO_3_ enhances endogenous bicarbonate buffering capacity by inducing significant, albeit transient, elevations in extracellular bicarbonate. Consequently, this enhances efflux of hydrogen cations (H^+^) from skeletal muscle, therefore delaying muscle fatigue and positively affecting numerous performance variables, such as power output [5] and time to exhaustion [6]. While it remains unclear whether minimal increases are required to achieve these benefits, substantial changes (~ 6 mmol∙L^−1^) in blood bicarbonate may improve the likelihood of performance-enhancing effects [2, 7]. Given that bicarbonate is lost in the neutralisation of gastric acid [8], large oral doses (200–300 mg kg^−1^ body mass) are required to induce meaningful elevations in the blood.

Acute gastrointestinal (GI) distress is a known side-effect of ingesting large amounts of NaHCO_3_ [9], particularly when administered as an aqueous solution [10]. Ergogenic effects have still been observed in those reporting GI distress [1, 11]; however, there is evidence to suggest that GI distress may be ergolytic for some individuals [1, 12–14]. Furthermore, some authors have suggested that GI distress may deter individuals from using NaHCO_3_ regardless of its potential ergogenic benefits [7]. Although the impact of GI distress on performance remains ambiguous, symptoms such as vomiting and diarrhoea may present a major practical limitation for athletes and coaches.

Polymeric-coated compounds can resist gastric degradation and reduce GI symptoms provoked by acid sensitive compounds, such as NaHCO_3_ [15]. Hydroxypropyl methylcellulose, contained in delayed-release capsules, can resist degradation in acidic environments (pH ~ 1–2 arbitrary units (AU)), and therefore, provides gastro-resistant properties. Instead, degradation occurs in the duodenum where the pH is far more alkaline (pH ~ 6–7 AU) and absorption can take place rapidly. Since GI distress is partly attributable to degradation in the stomach [8], it has been suggested that gastro-resistant capsules may alleviate symptoms that are typical with NaHCO_3_ ingestion [16]. Given that less bicarbonate is lost in the stomach, it has also been suggested that smaller doses may produce comparable acid-base changes to larger doses [16]. In contrast, as gut transit time is reduced with gastro-resistant formulations [15], this may reduce bicarbonate bioavailability when administered in this form. No study to date has examined the use of delayed-release NaHCO_3_ on markers of GI distress, nor on bicarbonate bioavailability and subsequent blood acid-base responses. Reducing GI distress following NaHCO_3_ ingestion may enhance use by athletes, particularly among those who are deterred by potential side-effects.

Therefore, the aim of this study was to investigate whether delayed-release NaHCO_3_ could mitigate GI distress compared with an aqueous solution, as well as to compare the pharmacokinetic and acid-base responses. It was hypothesised that delayed-release NaHCO_3_ would reduce GI symptoms and display at least bioequivalence when compared to an aqueous solution.

## Methods

### Participants

Twelve trained [17] healthy males (mean ± SD age 25.8 ± 4.5 years, maximal oxygen uptake ($\dot{\mathrm{V}}{\mathrm{O}}_{2\max }$) 58.9 ± 10.9 mL kg min^−1^, height 1.8 ± 0.1 m, body mass 82.3 ± 11.1 kg, fat-free mass 72.3 ± 10.0 kg) were recruited for the study. The study was approved by the University Research Ethics Committee (URESC) before the participants gave written informed consent to take part in the study. Inclusion in the study required that participants had performed regular (≥ 3 days week^−1^) physical exercise for at least 2 years. Exclusion criteria included ingestion of any buffering agents < 6 months prior to commencing the study and those with hypertension or on a sodium-restricted diet.

### Study Overview

Before taking part in the experimental trials, each participant underwent a baseline assessment over two laboratory visits separated by at least 48 h to establish (1) body composition and $\dot{\mathrm{V}}{\mathrm{O}}_{2\max }$ and (2) fluctuations in blood analytes (HCO_3_^−^, pH and base excess) under normal conditions. Fluctuations in blood analytes and GI symptoms under normal conditions were used as a control (CON) measure throughout. In the experimental trials, all participants underwent two conditions: 300 mg kg^−1^ body mass NaHCO_3_ administered as either an aqueous solution (SOL) or encased in delayed-release capsules (CAP). Experimental trials were administered in a block randomised crossover design that was counterbalanced (Latin square) in order of administration and took place at least 7 days apart to allow for the washout of residual NaHCO_3_ [18]. Participants were required to abstain from alcohol or caffeine-containing beverages for 12 h and from strenuous exercise 24 h before each laboratory visit. All sessions took place under standardised laboratory conditions (temperature = 21–22 °C, relative humidity = 50–55%, barometric pressure = 756–759 mmHg) and were conducted at 0900 h to account for circadian rhythms [19].

### Baseline Assessment

Participants arrived at the laboratory on both occasions after an overnight fast (~ 12 h) and euhydrated. On one visit, semi-nude body mass and fat-free mass were assessed using whole-body air displacement plethysmography (BOD POD®, COSMED, Italy). Participants then performed an incremental exercise test to volitional exhaustion on an electromagnetically braked cycle ergometer (Excalibur Sport, Lode, Netherlands). After a standardised 5-min warm-up at a power output of 70 watts (W), the cycling protocol commenced at 75 W for 1 min and workload increased by 1 W every 2 s (30 W min^−1^) until volitional exhaustion. This was determined by the inability of the participant to sustain their respective self-selected cadence for > 5 s despite feedback and strong verbal encouragement. On a separate visit, fingertip capillary blood samples were obtained using an aseptic technique after the participants were quietly seated for 20 min. Blood samples were drawn every 20 min over 180 min, with 10 min sampling between 80 and 140 min to accurately capture peak values [20]. Blood samples were collected in 100-μL heparin-coated glass clinitubes (Radiometer Medical Ltd., Denmark) and immediately analysed using a blood gas analyser (ABL800 BASIC, Radiometer Medical Ltd., Denmark). At the same time points, GI symptoms were recorded using a 9-item questionnaire, including nausea, flatulence, stomach cramping, belching, stomach ache, bowel urgency, diarrhoea, vomiting and stomach bloating [10]. Symptoms were self-measured on a 10-cm scale, the ends of which were marked “0, no symptom” and “10, severe symptom”, as previously described [11]. Participants remained seated throughout, although toilet breaks were permitted. No food was consumed during the experimental trials, and water was permitted ad libitum, with volumes replicated in the subsequent experimental session.

### Experimental Trials

Treatment condition SOL was prepared in 400 mL of natural mineral water (Evian®, France) and mixed with 50 mL of sugar-free blackcurrant flavoured squash (Robinsons®, UK) and refrigerated (~ 1 h) to enhance palatability [11]. For the CAP condition, size 00 capsules (DRcaps™, Capsugel®, France) were prefilled with NaHCO_3_ using a capsule filler (Capsule Connection LLC, USA), while doses were checked for accuracy using digital laboratory scales (Fisher, OHAUS™). Participants were instructed to ingest either the SOL or CAP with an equal volume (400 mL) of water within 10 min while the stopwatch commenced parallel with the start of ingestion [20, 21]. All experimental trials were conducted under the same conditions as the CON trial, and blood analytes and GI symptoms were measured as previously described.

### Statistical Analysis

Prospective statistical power analysis was conducted a priori to determine that 12 participants were required, with alpha and beta set at 0.05 and 0.20, respectively. Data were assessed for normality using standard graphical methods prior to analyses [22]. Two-way analysis of variance (ANOVA) with repeated measures (condition × time) was used to establish significant main effects for blood analytes (HCO_3_^−^, pH and BE) and GI symptom scores. Condition consisted of two levels (SOL and CAP), whereas time consisted of 13 (0, 20, 40, 60, 80, 90, 100, 110, 120, 130,140, 160 and 180 min). Effect sizes were calculated using partial eta squared (*η*^2^) for ANOVA and were interpreted according to Cohen [23] as follows: trivial < 0.20, small 0.20–0.49, moderate 0.50–0.79 and large ≥ 0.80. Blood analytes and GI symptom scores were then analysed using one-way ANOVA to establish differences at individual time points. Sphericity was assessed using Mauchly’s test throughout. Where appropriate, corrections for violations of sphericity (Greenhouse-Geisser) and multiple comparisons of differences within a factor (Bonferroni) were made [24]. Mean pharmacokinetic variables and highest GI symptom score between conditions were analysed by paired-sample *t* test. Descriptive data are presented as mean ± SD unless stated otherwise. The α-level of statistical significance was set at *P* < 0.05, and exact *P* values are given in the text and tables. Values for *P* of “0.000” given by the statistical package were corrected to “< 0.0005” [25]. Data were analysed using the Statistical Package for the Social Sciences (SPSS®) for Windows® (IBM, Chicago, IL, USA), version 25.

## Results

### Gastrointestinal Distress

**Fig. 1:**
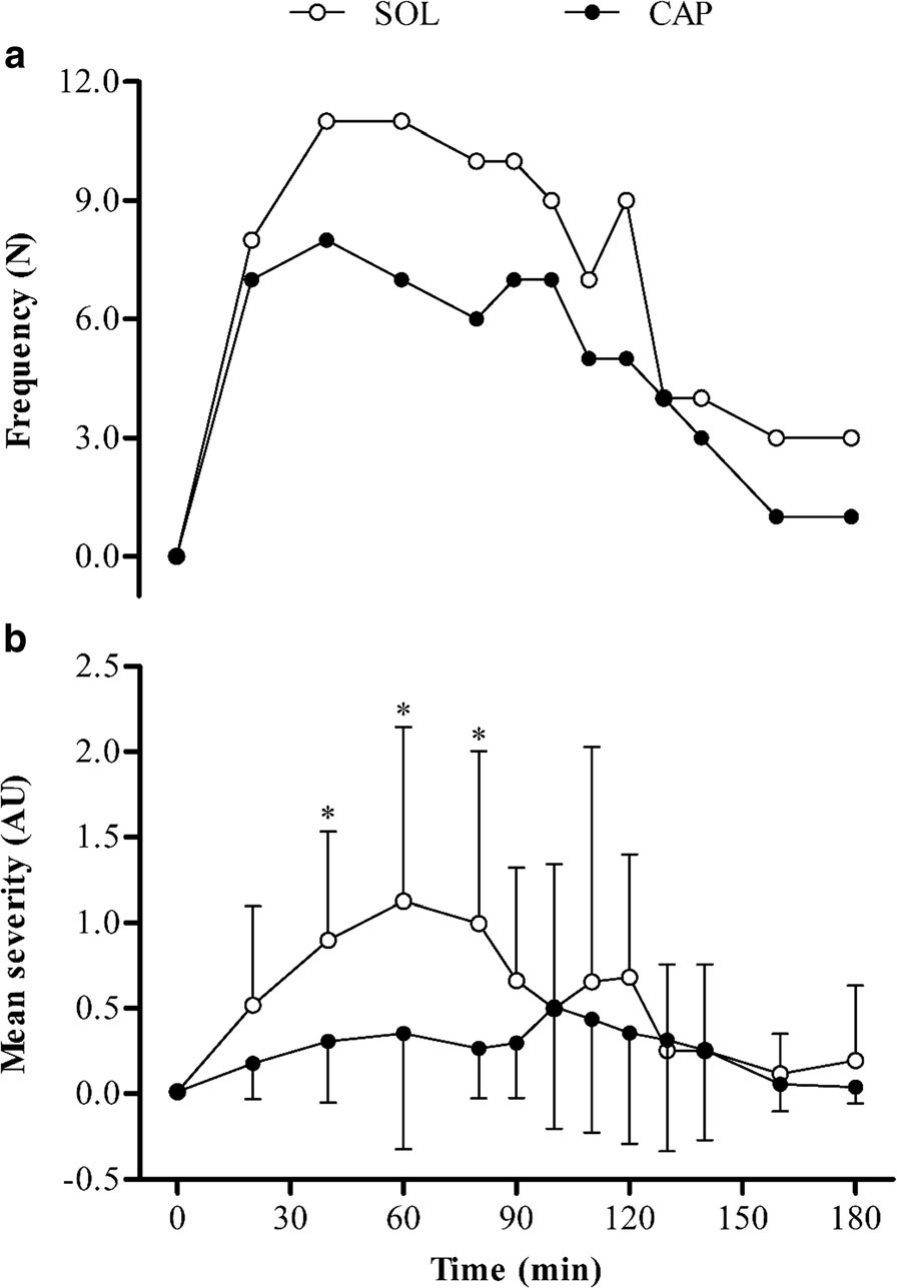
Incidence (**a**) and mean (±SD) severity (**b**) of GI symptoms. The asterisk denotes significant difference between SOL and CAP conditions (*P* < 0.05)

No GI symptoms were reported pre-ingestion, nor at any time point in the CON condition. All participants (*N* = 12) experienced at least one GI symptom following SOL and CAP ingestion (Table 1). Stomach bloating was the most prevalent GI symptom in both experimental trials, although this was lower with CAP (58%) than with SOL (100%). Overall, fewer GI symptoms (mean difference = 45.1%) were reported with CAP than with SOL (Fig. 1a). Incidences of GI distress peaked at 40 min post-ingestion under both conditions (Fig. 1a), which was predominantly due to belching and bowel urgency.

**Table 1:** The most severe individual GI symptom reported during any trial. Symptom scores are displayed in parenthesis and are expressed as arbitrary units (AU)

Participant	CON	SOL	CAP
1	Nil (0.0)	Stomach cramp (3.5)	Stomach bloating (3.0)
2	Nil (0.0)	Bowel urgency (7.0)	Stomach bloating (3.0)
3	Nil (0.0)	Nausea (6.0)	Nausea (2.0)
4	Nil (0.0)	Diarrhoea (10.0)	Stomach bloating (7.0)
5	Nil (0.0)	Diarrhoea (10.0)	Diarrhoea (7.0)
6	Nil (0.0)	Diarrhoea (10.0)	Diarrhoea (5.5)
7	Nil (0.0)	Bowel urgency (6.0)	Bowel urgency (5.0)
8	Nil (0.0)	Bowel urgency (10.0)	Bowel urgency (2.0)
9	Nil (0.0)	Diarrhoea (10.0)	Belching (3.0)
10	Nil (0.0)	Diarrhoea (10.0)	Belching (3.0)
11	Nil (0.0)	Stomach ache (3.0)	Belching (3.0)
12	Nil (0.0)	Diarrhoea (10.0)	Diarrhoea (7.0)
Mean (SD)	0.00 ± 0.00	7.96 ± 2.73 AU	4.21 ± 1.97 AU

Overall GI symptoms increased in the SOL (*P* < 0.0005) and CAP (*P* < 0.017) conditions beyond those observed in the CON condition. There was a significant effect of ingestion form (*F*_1.00, 11.00_ = 21.13, *P* = 0.001, *η*^2^ = 0.66), with less severe GI symptoms reported with CAP than with the SOL (*P* = 0.001) (Fig. 1b). There was no effect of time (*F*_2.85, 31.36_ = 2.89, *P* = 0.053, *η*^2^ = 0.21), although symptoms at 40 min were significantly greater than pre-ingestion (*P* = 0.03). No significant interaction was found (*F*_3.22, 35.39_ = 1.87, *P* = 0.148, *η*^2^ = 0.15). Overall GI symptoms were significantly greater with the SOL at 20 (*P* = 0.004), 40 (*P* < 0.0005), 60 (*P* = 0.002), 80 (*P* = 0.001), 90 (*P* = 0.002) and 120 (*P* = 0.018) min post-ingestion than in the CON condition. Symptoms were significantly lower at 40 (*P* = 0.004), 60 (*P* = 0.035) and 80 (*P* = 0.017) min post-ingestion with CAP than with the SOL. Gastric symptoms were significantly lower at 40 (*P* = 0.006), 60 (*P* = 0.020) and 80 (*P* = 0.021) min post-ingestion with CAP than with the SOL, while no significant differences were reported for intestinal symptoms (*P* > 0.05). There was a significant difference in the most severe GI symptom experienced in the SOL (7.21 ± 2.48 AU) and CAP (4.29 ± 2.12 AU) conditions (*P* = 0.002), respectively (Table 1). Time to reach the most severe individual GI symptom was greater with the SOL (87.50 ± 50.29 min) than with CAP (75.00 ± 32.33 min), although these were not significant (*P* > 0.05).

### Bicarbonate Bioavailability

**Fig. 2:**
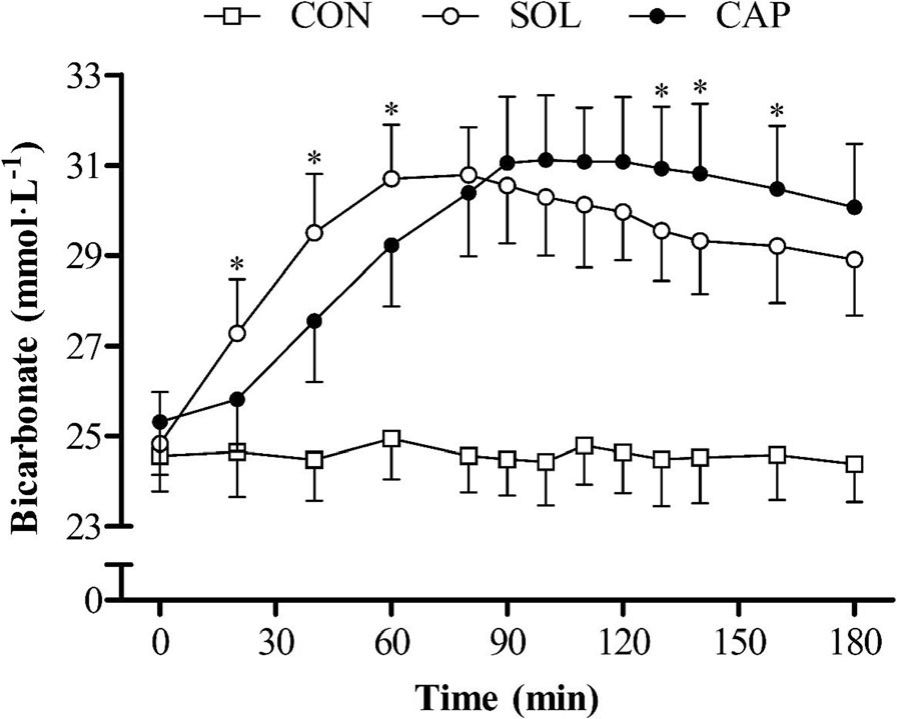
Mean (± SD) blood bicarbonate concentrations. The asterisk denotes significant difference between SOL and CAP conditions (*P* < 0.05)

**Fig. 3:**
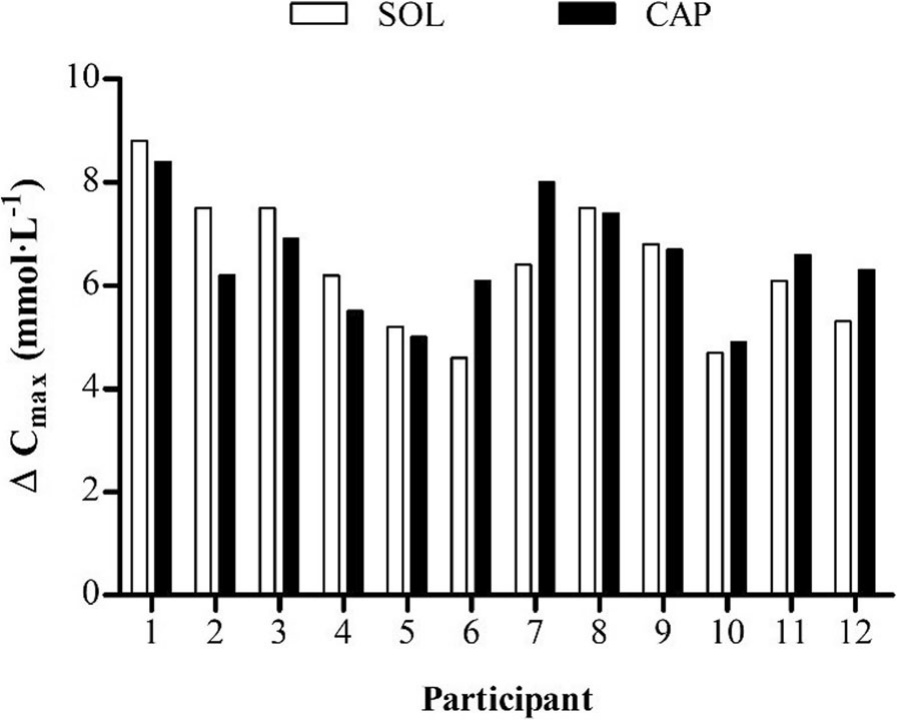
Individual changes in blood bicarbonate concentration between conditions (SOL and CAP)

Ingestion form had no significant effect on bicarbonate concentration (*F*_1.00, 11.00_ = 0.71, *P* > 0.05, *η*^2^ = 0.061) up to 180 min post-ingestion. There was a significant effect of time (*F*_2.38, 26.23_ = 101.74, *P* < 0.0005, *η*^2^ = 0.90); bicarbonate concentration increased notably for 60 min following ingestion of the SOL, until a decrease occurred from the previous time point at 180 min (*P* = 0.004) post-ingestion (Fig. 2). In the CAP condition, bicarbonate concentration rose progressively between 40 and 90 min, after which bicarbonate did not significantly change (*P* > 0.05). A significant interaction was found between condition and time (*F*_2.31, 25.44_ = 16.48, *P* < 0.0005, *η*^2^ = 0.60). Bicarbonate concentrations were significantly higher with the SOL at 20 (*P* = 0.008), 40 (*P* = 0.001) and 60 (*P* = 0.011) min post-ingestion than with the CAP and significantly lower at 130 (*P* = 0.021), 140 (*P* = 0.019) and 160 (*P* = 0.047) min post-ingestion. Mean pharmacokinetic variables were similar between conditions (Table 2). There was a delay in the absorption of bicarbonate with CAP; lag time (*T*_lag_) was greater with CAP than with SOL (*P* = 0.002), as was the time to reach peak bicarbonate concentration (*P* < 0.0005). Peak bicarbonate concentration (*C*_max_), change in bicarbonate concentration (∆*C*_max_) and area under the curve (AUC_0-3h_) increased in the SOL and CAP conditions (*P* < 0.005) compared with the CON, with no significant differences between conditions (*P* > 0.05). However, a greater number of participants reached a 5 mmol L^−1^ (SOL *N* = 10, CAP *N* = 11) and 6 mmol L^−1^ (SOL *N* = 8, CAP *N* = 9) increase in bicarbonate with CAP than with the SOL (Fig. 3).

**Table 2:** Mean (± SD) pharmacokinetic response variables for bicarbonate in the SOL and CAP conditions, together with the statistical significance of the difference

Outcome	SOL	CAP	*t* test	*P* value
*T*_lag_ (min)	20.0 ± 0.0*	31.7 ± 10.3*	− 3.92	0.002
*T*_max_ (min)	71.7 ± 18.0**	120.0 ± 28.9**	− 5.35	< 0.0005
*C*_max_ (mmol L^−1^)	31.2 ± 1.1	31.8 ± 1.3	− 1.66	0.125
∆*C*_max_ (mmol L^−1^)	6.4 ± 1.3	6.5 ± 1.1	− 0.46	0.658
AUC_0-3h_ (mmol min L^−1^)	5277.9 ± 173.9	5286.0 ± 197.9	− 0.13	0.899

The asterisk denotes significant difference between SOL and CAP (*P < 0.05, **P < 0.0005). *T*_*lag*_ time to commence change in bicarbonate concentration, *T*_*max*_ time to peak concentration, *C*_*max*_ peak bicarbonate concentration, *∆C*_*max*_ absolute change in bicarbonate concentration, *AUC*_*0-3h*_ area under the concentration-time curve

### Acid-Base Balance

**Fig. 4:**
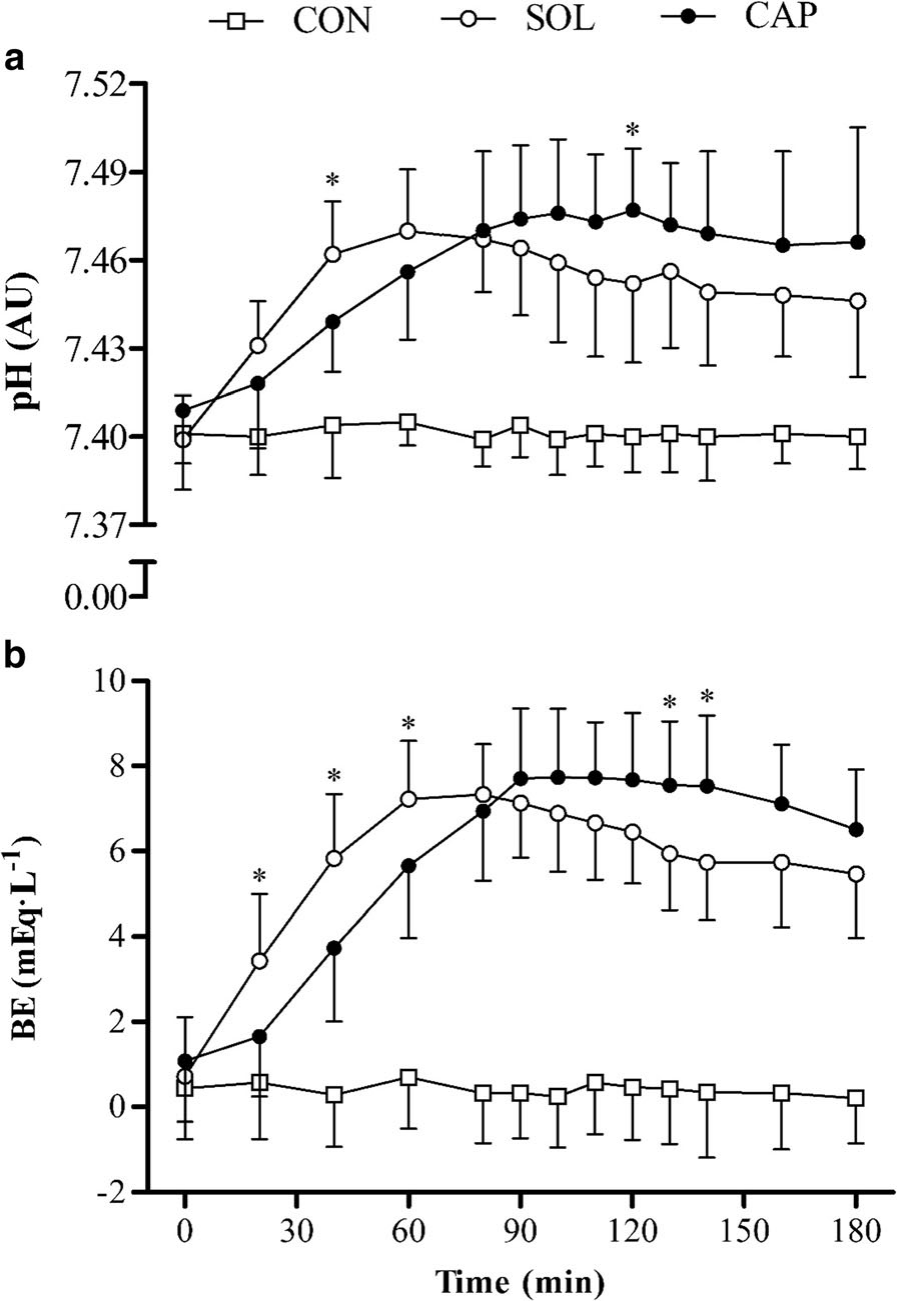
Mean (± SD) pH (**a**) and BE (**b**) responses. The asterisk denotes significant difference between SOL and CAP conditions (*P* < 0.05)

Ingestion form had no significant effect on pH (*F*_1.00, 11.00_ = 2.88, *P* > 0.05, *η*^2^ = 0.21) up to 180 min post-ingestion. There was a significant effect of time (*F*_4.42, 48.60_ = 43.74, *P* < 0.0005, *η*^2^ = 0.80); pH increased markedly for 60 min following ingestion of the SOL, until a decrease occurred from the previous time point at 180 min (*P* = 0.004) post-ingestion (Fig. 4). In the CAP condition, pH rose progressively between 40 and 90 min, after which pH did not significantly change (*P* > 0.05). A significant interaction was found between condition and time for pH (*F*_4.88, 53.67_ = 6.42, *P* < 0.0005, *η*^2^ = 0.37). Blood pH was significantly higher with the SOL at 40 min (*P* = 0.009) post-ingestion than with the CAP and significantly lower at 120 min (*P* = 0.017) post-ingestion. Blood pH peaked much later with the CAP (SOL = 71.67 ± 25.88 min, CAP = 125.83 ± 27.75 min, *P* = 0.001) than with the SOL, although absolute changes were comparable between conditions (*P* = 0.093).

Similarly, ingestion form had no significant effect on base excess (BE) (*F*_1.00, 11.00_ = 0.69, *P* > 0.05, *η*^2^ = 0.06) up to 180 min post-ingestion. There was a significant effect of time (*F*_2.24, 24.68_ = 118.08, *P* < 0.0005, *η*^2^ = 0.92); BE increased markedly for 60 min following ingestion of the SOL, until a decrease occurred from the previous time point at 180 min (*P* = 0.034) post-ingestion (Fig. 4). In contrast, BE rose progressively between 40 and 90 min in the CAP condition, after which BE did not significantly change (*P* > 0.05). A significant interaction was found between condition and time (*F*_2.20, 24.18_ = 15.35, *P* < 0.0005, *η*^2^ = 0.58). Blood BE was significantly higher with the SOL at 20 (*P* = 0.014), 40 (*P* = 0.005) and 60 (*P* = 0.034) min post-ingestion than with the CAP and significantly lower at 130 (*P* = 0.022) and 140 (*P* = 0.019) min post-ingestion. Blood BE peaked much later with CAP (SOL = 71.67 ± 18.01 min, CAP = 112.50 ± 27.01 min, *P* < 0.0005) than with the SOL, although absolute changes were comparable between conditions (*P* = 0.071).

## Discussion

This is the first study to investigate the effects of gastro-resistant capsules on GI distress, bicarbonate bioavailability and subsequent acid-base responses following NaHCO_3_ ingestion. The main finding was that delayed-release NaHCO_3_ mitigated GI distress, as hypothesised. Fewer GI symptoms (~ 45.1%) were reported with the delayed-release capsules, and the overall severity was reduced (~ 47.1%) when compared to the aqueous solution. Interestingly, reductions in GI symptoms were due to gastric but not intestinal symptoms, a finding that has been suggested in the relevant literature [15]. Gastrointestinal symptoms were negated with the delayed-release capsules, with a reduction in the most severe symptom experienced up to 3 h following supplementation (Table 1). Given that GI symptoms may be ergolytic [13, 14, 26], delayed-release NaHCO_3_ may be more ergogenic in those who experience severe GI distress with the aqueous solution. Furthermore, since GI distress may deter some individuals from using NaHCO_3_ as an ergogenic aid [7, 10], delayed-release NaHCO_3_ would appear to be a more favourable option for athletes and coaches.

While necessary to achieve erogenicity [27], large boluses (~ 200–300 mg kg^−1^ body mass) of NaHCO_3_ can induce significant GI symptoms. In the current study, there was a high incidence of GI distress with the aqueous solution, which is in agreement with some authors [13] but not others [10, 28]. Symptoms are considered to have both gastric and intestinal causes [8], a finding that is supported by the current study. On entering the stomach, NaHCO_3_ dissociates to sodium and bicarbonate ions, the latter of which produces carbon dioxide during the neutralisation of gastric acid [8]. Consequently, carbon dioxide tension increases exponentially with exposure and is associated with gastric symptoms, such as belching, nausea and stomach ache. Intestinal symptoms, though partly induced from elevated carbon dioxide tension in the intestinal lumen, originate from excess sodium that aggravates the intestinal mucosa and creates osmotic fluctuations leading to bowel urgency and diarrhoea [13]. Delayed-release capsules, partly formulated with a polymeric barrier, have gastro-resistant properties and can minimise disintegration in the stomach. Mitigating gastric symptoms may indeed have implications for performance. Previous research indicates that symptoms can inhibit high-intensity cycling performance [14], while others have reported improvements irrespectively [26]. Since numerous participants have withdrawn from studies due to GI distress [29], previous research may have underestimated the ergolytic effect of such symptoms. Studies that have attempted to mitigate GI symptoms following NaHCO_3_ ingestion have done so using alternative dosing strategies. Gelatine capsules co-ingested with a small high-carbohydrate (1.5 g kg^−1^ body mass) meal are currently regarded as the formulation least likely to induce GI symptoms following NaHCO_3_ ingestion [10]. In the current study, delayed-release capsules were ingested after an overnight fast, largely to minimise potential confounding effects of food on acid-base changes. Nevertheless, co-ingestion with a small high-carbohydrate meal may have further reduced GI symptoms and warrants further investigation. Furthermore, while comparison with an aqueous solution was chosen based on its frequency of use within the literature, this may not be the case in the practice and is thus a limitation to the study. Future work should look to assess the pharmacokinetics of NaHCO_3_ administered based on capsule composition only, so that the mechanism underpinning bioavailability and reductions in GI distress may be better understood.

In relation to bioavailability, both ingestion forms provided adequate sources of bicarbonate and displayed similar pharmacokinetic properties. Increases in bicarbonate were comparable, with both forms exceeding the 6 mmol L^−1^ threshold suggested to enhance ergogenicity [2]. Interestingly, some (*N* = 3) participants displayed enhanced bicarbonate availability (≥ 1 mmol L^−1^) with delayed-release capsules (Fig. 4), while only one participant was found to have enhanced bicarbonate availability of this magnitude with the aqueous solution. Similarly, more participants achieved a 5 mmol L^−1^ (SOL *N* = 10, CAP *N* = 11) and 6 mmol L^−1^ (SOL: *N* = 8; CAP: *N* = 9) increase in bicarbonate with the delayed-release capsules than with the aqueous solution. These results would be explained by the gastric bypass model proposed by Oliveira et al. [16], which includes the effect of gastric transit time, and bicarbonate loss associated with neutralisation. As suggested by these authors, reducing bicarbonate neutralisation in the stomach increases bioavailability when NaHCO_3_ is administered orally. Since changes of ~ 1 mmol L^−1^ in bicarbonate concentration can positively affect performance [26], delayed-release NaHCO_3_ may elicit superior ergogenicity. In contrast to the aqueous solution, bicarbonate absorption did not commence immediately following capsule ingestion, suggesting that the delayed-release capsules were effective [15, 30]. This result indicates that the capsules achieved disintegration in the intestine, which had the effect of lengthening (+ 48.3 min) the time to reach peak bicarbonate concentration. Bicarbonate peaked at ~ 120 min post-ingestion with the delayed-release capsules, which is later than previously reported with an aqueous solution in some studies [20] but not all [21]. Similar to previous studies [31–33], there was a high degree of individual variability in the time to reach peak bicarbonate concentration, although this was greater with the capsules. The current findings for bicarbonate indicate that for most individuals, delayed-release NaHCO_3_ may increase the likelihood of inducing a performance-enhancing effect; however, as this was not consistent for all individuals, decisions around ingestion form should be based upon individual concentration-time profiles.

Metabolic alkalosis was induced earlier with the aqueous solution (~ 40 min) than with delayed-release capsules (~ 60 min), although this state was maintained for longer (+ 30 min) when ingested in the delayed-release form. Homeostatic regulation, through respiratory compensation [34], may have been stimulated to a lesser extent with slower bicarbonate absorption, rather than the abrupt elevation observed with an aqueous solution. Exercise performance timed with peak alkalosis may enhance the ergogenicity of NaHCO_3_ [7, 29]; therefore, it is reasonable to consider that delayed-release may provide a larger ergogenic window. In a competitive setting, this may be more favourable since performance may not commence parallel with peak alkalosis due to variable factors, such as sports fixtures. In the current study, time to reach peak alkalosis was much later with delayed-release NaHCO_3_ (~ 125 min) than with the aqueous solution (~ 72 min), with one participant peaking at 180 min post-ingestion. In a practical setting, delayed-release NaHCO_3_ would have to be ingested sooner than an aqueous solution to elicit similar acid-base changes. This may be favourable in terms of ergogenicity since GI symptoms were negligible at later time points. In contrast, given that bicarbonate concentrations were significantly lower with delayed-release NaHCO_3_ up to 60 min post-ingestion, this form may be less ergogenic when ingested less than 60 min prior to exercise.

## Conclusions

In summary, delayed-release NaHCO_3_ mitigates GI symptoms and these effects do not reflect the intestinal component but rather the gastric component of overall symptoms. The similar pharmacokinetic properties, coupled with a delay in the time to reach metabolic alkalosis, mean that delayed-release NaHCO_3_ requires earlier ingestion than with an aqueous solution to induce comparable acid-base changes. The current study supports the gastric bypass model, which can be used as a model for exploring various ingestion forms and modes of administration generally. Lastly, delayed-release NaHCO_3_ may be more ergogenic in those who experience severe gastrointestinal distress with an aqueous solution, although this warrants further investigation.

## Abbreviations

∆*C*_max_: Absolute change in bicarbonate concentration
AUC_0-3h_: Area under the concentration-time curve
CAP: Delayed-release sodium bicarbonate
*C*_max_: Peak bicarbonate concentration
CON: Control
NaHCO_3_: Sodium bicarbonate
SOL: Aqueous solution sodium bicarbonate
*T*_lag_: Time to commence change in bicarbonate concentration
*T*_max_: Time to peak bicarbonate concentration
